# Alzheimer’s Disease and Alzheimer’s Disease-Related Dementias in African Americans: Focus on Caregivers

**DOI:** 10.3390/healthcare11060868

**Published:** 2023-03-16

**Authors:** Jonathan Kopel, Ujala Sehar, Moumita Choudhury, P. Hemachandra Reddy

**Affiliations:** 1 Department of Internal Medicine, Texas Tech University Health Sciences Center, Lubbock, TX 79430, USA; 2 Department of Speech, Language and Hearing Sciences, School Health Professions, Texas Tech University Health Sciences Center, Lubbock, TX 79430, USA; 3 Department of Public Health, School of Population and Public Health, Texas Tech University Health Sciences Center, Lubbock, TX 79430, USA; 4 Neurology, Departments of School of Medicine, Texas Tech University Health Sciences Center, Lubbock, TX 79430, USA; 5 Nutritional Sciences Department, College of Human Sciences, Texas Tech University, Lubbock, TX 79409, USA

**Keywords:** African Americans, dementia, Alzheimer’s disease, disparity, socioeconomic, healthcare

## Abstract

Alzheimer’s disease (AD) and Alzheimer’s Disease-Related Dementias (ADRD) are chronic illnesses that are highly prevalent in African Americans (AA). AD and ADRD are caused by multiple factors, such as genetic mutations, modifiable and non-modifiable risk factors, and lifestyle. Histopathological, morphological, and cellular studies revealed how multiple cellular changes are implicated in AD and ADRD, including synaptic damage, inflammatory responses, hormonal imbalance, mitochondrial abnormalities, and neuronal loss, in addition to the accumulation of amyloid beta and phosphorylated tau in the brain. The contributions of race, ethnicity, location and socioeconomic status all have a significant impact on the care and support services available to dementia patients. Furthermore, disparities in health care are entangled with social, economic, and environmental variables that perpetuate disadvantages among different groups, particularly African Americans. As such, it remains important to understand how various racial and ethnic groups perceive, access, and experience health care. Considering that the mounting data shows AA may be more susceptible to AD than white people, the demographic transition creates significant hurdles in providing adequate care from family caregivers. Furthermore, there is growing recognition that AD and ADRD pose a significant stress on AA caregivers compared to white people. In this review, we examine the current literature on racial disparities in AD and ADRD, particularly concerning AA caregivers.

## 1. Introduction

Alzheimer’s disease (AD) and Alzheimer’s Disease-Related Dementias (ADRD) are rising global healthcare concerns that largely affect the elderly. It involves a collection of symptoms collectively referred to as dementia that negatively affect memory, thinking, and social skills to the point where they interfere with daily living. Alzheimer’s disease, vascular dementia, Lewy body dementia, frontotemporal dementia, mixed dementia, and other dementias can all contribute to it and are termed Alzheimer’s Disease-Related Dementias. The most prevalent type of dementia is Alzheimer’s disease. Memory loss and severe cognitive deficits are hallmarks of Alzheimer’s disease (AD), which is the fourth greatest cause of death worldwide for the elderly [1]. Although a definitive etiology of AD/ADRD is yet unknown, both genetic factors, such as Amyloid-beta precursor protein (APP), Presenilin-1 (PS1), Presenilin-2 (PS2) genes, and environmental factors (e.g., volatile anesthetics, toxic metals, industrial chemicals, air pollutants, and pesticides) are believed to be involved [2]. In late-onset AD, apolipoprotein E (APOE), particularly the APOE*4 variant, has been identified as a potent susceptibility marker that contributes to almost 30% of the risk [3]. In addition, other pre-existing medical conditions, such as diabetes, cerebrovascular disease, and hypertension, as well as cognitive activity, physical activity, and lifestyle choices, all contribute to the onset and pathogenesis of AD/ADRD [2]. The causes of AD/ADRD are thought to be both genetic and non-genetic, although the exact cause of the illness is yet unknown [4]. Alzheimer’s disease causes the progressive malfunction and death of neurons, which leads to gradual memory loss and impairments in cognitive skills [5,6]. Among the different types of memory, AD affects both implicit and explicit memory, which interferes with a patient’s ability to recall information or events [7]. The early clinical signs of AD are memory impairment, although cognitive impairments and personality changes are commonly observed in AD patients [8]. In addition, AD can exist in two different forms: familial and late-onset AD. Early-onset AD patients exhibit a quick progression in cognitive deterioration, whereas late-onset AD patients experience more memory disruption [9].

**Figure 1:**
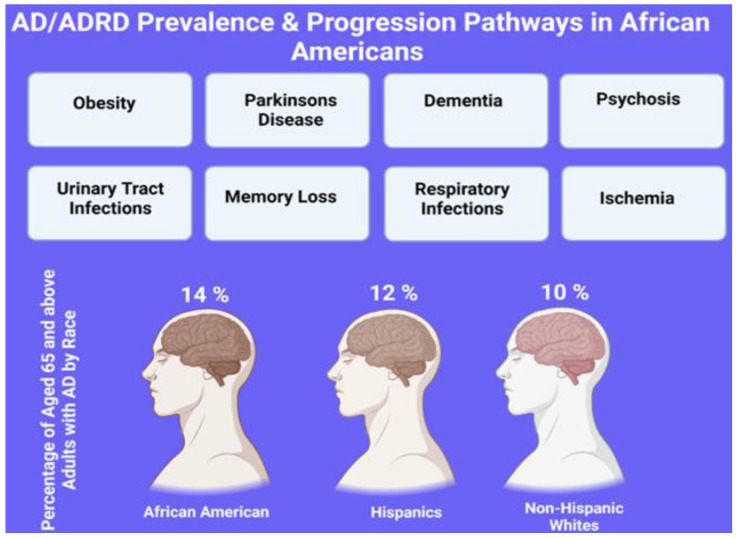
Prevalence and progression pathways of Alzheimer’s disease and related dementias in African Americans.

The contributions of race, ethnicity, location and socioeconomic status all have a significant impact on the care and support services available for AD/ADRD patients [10]. Furthermore, disparities in health care are entangled with social, economic, and environmental variables that perpetuate disadvantages among different groups, particularly African Americans (AA), concerning caregiving and other support services [10]. Considering the mounting data showing that AA may be more susceptible than AD in white people, referring to Figure 1, the coming demographic transition in the United States (U.S.) creates significant hurdles in providing adequate care from family caregivers.

In this review, we examine the current literature on racial disparities in AD/ADRD, particularly concerning AA caregivers. First, we examine the pathophysiology of AD with a particular focus on the unique biological, environmental, and social factors that influence the onset and progress of AD in AA. Next, we examine the unique challenges AA caregivers encounter when caring for their family member with AD/ADRD compared to other racial and ethnic groups paying particular attention to the historical and cultural influences on caregiver dynamics. Lastly, we explore the medical and social interventions that may improve caregiver burnout among AA caregivers [11].

### 1.1. Amyloid Beta (Aβ)

Amyloid beta is the cleavage product of the glycoprotein APP and is normally present in the brain as part of the signal transduction process [12,13,14,15]. The dysregulation of Aβ levels in the brain results in senile plaque development and Aβ deposition, both of which impair cognition in AD patients [12,13,14,15]. Three domains make up the transmembrane protein known as APP, two of which are found within cells and one of which is found on the cell membrane [12,13,14,15]. The α, β, and γ secretases break down the APP domains [12,13,14,15]. Different lengths of Aβ peptides, such as Aβ_42_ and Aβ_40_, are produced when APP is first cleaved by β-secretase and then further cleaved by γ-secretase [12,13,14,15]. The number of residues between these two main isoforms of Aβ differs because Aβ_42_ has two more residues at its C-terminus than Aβ_40_. In addition, the Aβ isoform 42 makes up most amyloid plaques in AD [12,13,14,15].

Due to an imbalance between the creation and clearance of these peptides from the different regions of the brain, the insoluble Aβ slowly builds up throughout a patient’s lifetime before AD symptoms develop. Most cases of AD are sporadic or due to ineffective Aβ-peptide elimination from the brain [16,17,18]. Familial AD, which is less frequent, is caused by changes in genes related to Aβ-metabolism. Mutations in the APP and presenilin genes, both of which are involved in Aβ metabolism, are the cause of early-onset, familial AD [16,17,18]. Similar to sporadic AD, neuronal loss and tissue atrophy, neurofibrillary tangles, and elevated levels of Aβ plaques are also present in familial AD [19,20,21,22]. Missense mutations in APP, PS1, and PS2 are the root cause of familial AD [19,20,21,22]. Missense mutations in APP are initially identified as the first genetic component contributing to AD [19,20,21,22]. The mutations in APP are located after the α-secretase site, before the β-secretase cleavage site, or the carboxyl-terminal of the γ-secretase cleavage site [19,20,21,22]. Despite the investigation, no further APP variants were discovered to cause AD, demonstrating that these missense mutations affect how three secretases process APP during its proteolytic processing, which ultimately leads to the progression and onset of AD [19,20,21,22]. Even though there are numerous mechanisms that contribute to the development of AD, the Aβ buildup remains one of the most popular theories [22]. The correlation between APP/PSI/PS2 mutations and Aβ synthesis and processing further supports the idea that Aβ plays a more important role in familial AD than in sporadic AD [22]. Globally, sporadic AD is the most prevalent form of dementia [23,24]. Large-scale genome-wide investigations have shown a variety of genetic variables that contribute to the multifactorial nature of the illness [23,24]. One of the risk factors for developing sporadic AD is aging itself [23,24]. Intracellular Aβ peptides set off a cascade of pathogenic processes, including oxidative stress, inflammation, synaptic dysfunction, mitochondrial malfunction, loss of calcium control, and synaptic dysfunction [23,24]. All these pathogenic processes in both sporadic and familial AD contribute to the gradual degeneration of neurons.

### 1.2. Tau Protein

Tau protein is a microtubule-associated protein that is primarily responsible for maintaining the stability of microtubules in axons and has important roles in synaptic plasticity, the regulation of genomic stability, and cell signaling [6,25]. The microtubule-associated protein (MAP) tau, which is particularly prevalent in the axons of neurons, stabilizes microtubules [26]. The adult human brain expresses six tau isoforms, all of which are generated from the same gene through alternative splicing [26]. The isoforms, which are referred to as 3R or 4R tau isoforms, might vary from one another in the quantity of tubulin-binding domains [27]. The presence or absence of one or two 29-amino-acid-long, acidic amino acids at the protein’s *N*-terminus can also modify the protein and its function [27]. A fundamental proline-rich area may be found between the projection and the microtubule-binding domains [27]. Despite the six isoforms’ apparent functional similarity, each is believed to play specific and, to a certain extent, unique physiological roles [28]. The different isoforms seem to manifest themselves differently during development [28]. In general, Tau proteins interact with tubulin to encourage the protein’s assembly into microtubules, helping to stabilize the structure of these structures. In addition, tau proteins modulate the anterograde transport of the microtubule-dependent axonal transport of organelles by interacting with kinesin and the dynein-driven retrograde transport.

Tau-mediated neurodegeneration is most likely caused by abnormal tau disengagement from the microtubules, which leads to a corresponding rise in tau concentration within the cytoplasm [28]. An imbalance of tau kinases and/or phosphatases, tau gene mutations, and the covalent/posttranslational modification of tau are some of the direct causes of aberrant tau disengagement from the microtubules [28]. Once tau is released from the microtubules, misfolding becomes more probable, with higher cytosolic tau concentrations increasing the likelihood of misfolding [28].

Subsequently, a structural transition leads to this more organized aggregate, and the eventual development of neurofibrillary tangles may form from several interactions with membranous structures [28]. Pathogenic tau proteins that are misfolded serve as a nidus once absorbed, drawing soluble endogenous tau into more distorted conformations that progressively spread throughout linked brain areas [29]. In AD, layer II of the entorhinal cortex serves as the starting point for the creation of NFTs, which then move via the limbic and associative regions until they reach the hippocampus and neocortex [30]. Thus, the spread of phosphorylated tau can spread from cell to cell, affecting the entire brain [30]. The spreading of tau between neurons and adjacent brain regions is a complex process involving several processes, such as the degradation, secretion, transmission, and toxicity of tau proteins [29]. Similar to Aβ and AD, the exact mechanism underlying the spread of tau pathology remains an area of active investigation [29].

### 1.3. Mitochondria and AD/ADRD

Due to its large lipid content, relatively high oxygen consumption, and low levels of antioxidant defenses, the brain is particularly susceptible to oxidative stress [31,32,33,34,35,36]. The mitochondrial electron transport chain (ETC) complexes I and III, as well as the TCA cycles of a-ketoglutarate dehydrogenase, are believed to be the sources of superoxide radicals [37,38]. Protein oxidation in the cytoplasm may result from the transport of H_2_O_2_ and superoxide radicals from the mitochondrial matrix and the inner and outer mitochondrial membranes [31,32,33,34,35,36]. To meet cellular energy requirements, synaptic mitochondria are first created in the cell bodies of neurons and are then transferred from the axon or dendrite [31,32,33,34,35,36]. This process is referred to as mitochondrial trafficking [31,32,33,34,35,36]. Natural mitochondrial trafficking may transfer injured or degraded mitochondria from the cell body to synaptic terminals [31,32,33,34,35,36]. For a variety of synaptic processes, such as the synaptic transmission of neurotransmitter exocytosis, the potentiation of neurotransmitter release, and synaptic development, synaptic terminals need large quantities of cellular ATP [31,32,33,34,35,36]. Furthermore, mitochondria are necessary for synaptic terminals to isolate and release Ca^2+^ for post-tetanic potentiation [31,32,33,34,35,36]. Transporting to synaptic terminals requires more efficient mitochondria. As a result of the transport, synaptic mitochondria may be older than cell-body mitochondria, and oxidative stress may cause more harm to them [31,32,33,34,35,36]. It is believed that the increased synaptic mitochondrial damage in AD/ADRD patients affects neurotransmission and ultimately results in a cognitive loss.

In the brain, the mitochondria of AD patients and transgenic mice have shown oxidative stress, which induces abnormalities in neuronal and astrocytic mitochondria. This suggests that neurons and astrocytes were damaged by free radicals released from mitochondria [31,32,33,34,35,36]. The brains of AD patients were also found to have lower amounts of three mitochondrial enzymes: pyruvate dehydrogenase complex, cytochrome oxidase, and alpha-ketoglutarate dehydrogenase complex [38]. Beyond free radical damage from mitochondria, the disruption to the mitophagy process in mitochondria could be associated with the development of AD.

### 1.4. Mitophagy and AD/ADRD

The deliberate destruction of mitochondria by autophagy is known as mitophagy. Following injury or stress, mitophagy eliminates dysfunctional mitochondria [39]. To allow for the orderly breakdown and recycling of damaged, old, or broken mitochondria, mitophagy requires a special membrane trafficking pathway [39]. Special cell cycle machinery removes the damaged or aging mitochondria from the cell cycle controls [39]. Mitophagy has several physiological functions in the cell, including encouraging mitochondrial turnover and employing lysosomes as well as autophagosomes with adaptor proteins to engulf damaged mitochondria [39].

As such, mitophagy avoids the buildup of dysfunctional mitochondria, ensuring mitochondrial quality to prevent cellular aging and removing damaged mitochondria [39].

Many neurodegenerative disorders, such as Alzheimer’s disease, are characterized by changes in mitophagy, which decrease their ability to remove damaged mitochondria [40]. When compared to the healthy controls, it has been observed that mitophagy is significantly lower in AD patients. Therefore, it is hypothesized that inhibiting mitophagy can cause a buildup of defective neurons in AD [40]. A crucial stage in the process of mitophagy is the fusion of mitochondria harboring autophagosomes with lysosomes, and the aberrant buildup of autophagosomal vacuoles in the neuronal cell bodies is frequently observed in AD [40]. One such protein, known as PARKIN, is an E3 ubiquitin ligase required to initiate mitochondrial mitophagy [40]. Recent studies have shown that PARKIN levels were lower in the cytoplasm of AD patient brains, along with abnormal PINK1 accumulation, leading to defective mitophagy [40]. The most significant factor contributing to the development of AD is the decrease in PINK1 and PARKIN, which increases the number of dysfunctional mitochondria as a result of impaired mitophagy. By producing reactive oxygen species (ROS) and interleukin, these damaged mitochondria cause oxidative stress and inflammation [40]. Since the balance between mitochondrial synthesis and degradation is essential for maintaining the quality control of the mitochondria, any disruption can result in the buildup of defective mitochondria and exacerbate the pathophysiology associated with AD [40]. 

## 2. Alzheimer’s Disease and Alzheimer’s Disease Related Dementias

Alzheimer’s disease begins as an episodic memory disorder and progresses to include problems with praxis, visuospatial orientation, language, math, and executive processes [41]. The evolution of deficits corresponds to the sequential involvement of several brain regions, starting with hippocampal injury and moving on to lateral temporal regions, parieto-occipital cortex, and frontal lobe components [41]. However, AD pathology is sometimes discovered in patients exhibiting a variety of clinical symptoms that are first produced by damage to less frequently affected cortical areas [41], as shown in Table 1. These unusual instances include Frontotemporal Dementia (FTD), Lewy-body Dementia (LBD), Multiple Etiology Dementia (MED), and Vascular Dementia (VD). The prevalence of AD atypical variants is unknown. Clinical problems covered by FTD include those that affect behavior, language, executive function, and motor symptoms [42]. According to the National Institute of Neurological Disorders and Stroke, LBD affects an estimated 1 million Americans and has symptoms, including progressive dementia plus various combinations of fluctuating cognition, visual hallucinations, mood disorders, and autonomic dysfunction. Whereas, in mixed/multiple etiology dementia, it may be difficult to determine how many of a person’s symptoms are brought on by AD or some other type of dementia. Vascular dementia is characterized as dementia that affects the brain’s blood supply. Numerous clinical issues (such as stroke) or ischemic brain damage to the white matter fibers in the brain might result from these blood vessel anomalies [43]. Early signs of different types of dementia can occasionally be difficult to diagnose as they can be vague and misleading. It may take several years before sufficient symptoms emerge to identify a particular form of dementia. A recent study looked at blood-based biomarkers—amyloid beta [A]_1-42/1-40_, neurofilament light [NfL], phosphorylated tau [p-tau]181, and glial fibrillary acidic protein [GFAP] that can distinguish between dementia caused by Alzheimer’s disease (AD), frontotemporal dementia (FTD), and dementia with LBD and found that a combination of plasma biomarkers NfL, p-tau181, and GFAP might be useful to discriminate AD, LBD, and FTD [44].

Especially in the brains of older people, vascular pathologies such as macro and micro infarcts, small and large vessel cerebrovascular disease, microbleeds, and white matter disease are prevalent. These pathologies lower the dementia threshold and cause cognitive impairment [49]. The pathophysiologic process of AD, which results in reduced cerebral blood flow, and AD-related dementia may also be influenced by vascular dysfunction, abnormalities in the blood–brain barrier, and other factors [49]. Due to the elevated risk that metabolic disorders pose for accelerating the progression of AD, consuming a diet rich in fat, glucose, or cholesterol may cause metabolic dysfunction, which can increase the risk of AD later in life. However, there is still debate regarding how lipids are metabolized in the brain and their precise impact on the brain or the pathology of AD [50]. The layers of myelin sheaths enclosing the brain and the spinal cord’s fibers may be the connection between fat and the central nervous system’s lipid content. In the mature PFC, there are also myelinating oligodendrocytes [51]. In the study of AD, some studies said that hyperlipidemia aggravates Aβ deposition and Tau phosphorylation due to worsening oxidative stress [52]. Others have stated that the ketogenic diet may avoid Alzheimer’s disease neurodegeneration and that hyperlipidemia improves the pathology of the disease [53]. 

In the study by Galton et al., atypical AD cases represent 14% of all AD cases [54]. Patients with atypical AD present in two primary ways affecting either the visuospatial or language-related structures in the brain [41]. Specifically, the visuospatial regions of the occipital and parietal cortices are dysfunctional in the posterior cortical atrophy (PCA) condition, while language-related structures are nearly entirely impacted by primary progressive aphasia (PPA) [41]. First described by Benson et al., the early onset (before the age of 60) of alexia with agraphia, Balint’s syndrome (optic ataxia, ocular apraxia, simultanagnosia, and visual agnosia), Gerstmann syndrome, and acalculia are key components of the PCA syndrome [54,55,56,57,58]. AD, dementia with Lewy bodies, frontotemporal dementia, and dementia secondary to diseases, including AIDS dementia, are currently the different types of dementia that are recognized. These atypical dementias differ from AD in that they frequently involve neurological symptoms, which reflect the localization of the degenerative process rather than the underlying histopathology [59].

Primary progressive aphasia was first described by Mesulam et al. as a syndrome consisting of the progressive deterioration of language and preservation of other cognitive functions, which was associated with left temporal and frontal lobe atrophy [41]. As these cases were primarily associated with the Frontotemporal Lewy body Dementia (FTLD) spectrum of diseases, the existence of early language deficits was usually considered an exclusion criterion for AD [41]. Further investigation showed that AD could underlie a higher percentage of PPA cases than was first believed. The syndrome of primary aphasia was classically divided into two distinct patterns: non-fluent aphasia and semantic dementia. Approximately, between 20 and 30% of PPA patients may have AD pathology. 

Another symptom of PPA is difficulty repeating sentences and comprehending lengthy passages while still being able to grasp single words. Imaging and functional investigations have connected this kind of PPA, known as logopedic aphasia, to the atrophy of the left temporoparietal junction, particularly in the left posterior superior and middle temporal gyri and inferior parietal lobule [60]. Clinical-pathological studies and imaging studies using the Pittsburgh compound B have shown that AD pathology appears to be common in AD patients with language disorders, although atypical Alzheimer’s cases rarely present with semantic and non-fluent aphasia [41]. Although dementia remains a growing healthcare issue, the AA is particularly at risk for dementia and caregiving burnout. In the following sections, we explore both areas to provide further context and insight into this growing healthcare crisis in the AA community.

## 3. African Americans—Origins and Background

Race has been and continues to be a ubiquitous problem in the American healthcare system, particularly for AA patients and dementia care [61]. African Americans have their heritage in sub-Saharan Africa. In general, those who were born in the United States (US), yet are of African descent, are referred to as AA [61,62]. In this review, we will be using the definition of AA or black as defined by the U.S. Census Bureau (Table 2). Most AAs today are descended from slaves, with a majority coming from West/Central Africa, where a large proportion of the transatlantic slave trade occurred [61,62]. West Africans were sold to European slave dealers in the 16th century and transported over the Atlantic to the Thirteen Colonies, which classically marked the beginning of AA history [61,62,63]. After being enslaved, many AAs were forced into labor on plantations, especially in the southern colonies. A select number were able to escape or become free to establish independent settlements both before and during the American Revolution. It was only after the Civil War that over four million AA slaves were freed. After slavery, AA obtained citizenship and the ability to vote.

However, due to political instability and the persistence of White Supremacist attitudes through Jim Crow Laws (1877–1954) as well as other state and local policies, AAs were demoted to second-class citizens and, in some cases, were subjected to bondage similar to when they were slaves. It was during this period that AAs also lost their voting rights in the South [61,62,63]. Jim Crow laws, which were a continuation of the segregation that started with slavery, were designed to force AAs to follow specific actions regarding their movement, speech, and even where they could eat, drink, or rest. For example, AAs had to wait until all White clients were attended to in racially mixed establishments [64]. To prevent violence, or retaliation from white people, the majority of AA complied with the Jim Crow legislation [64]. In the later part of the 19th century, Jim Crow and other discriminatory laws against AA increased [64]. These discriminatory acts included racial segregation, a lack of voting rights, the denial of economic opportunities or resources, and individual and mass acts of racial violence directed at AAs that went unchecked or were encouraged by government authorities [64].

During the first part of the 20th century, AAs in the South lived in appalling conditions, which caused many AAs to migrate North in what became known as the Great Migration [65]. The resulting migration to North cities led to a burgeoning African American population in both the Northern and Western regions of the US [65,66,67]. With growing AA communities in Northern and Western cities, the animosity between AAs and White people increased, leading to civil unrest and riots [65,66,67]. Despite moving away from the south, institutional discrimination against Blacks remained prevalent in both Northern and Western cities. This impacted many facets of daily life for Aas by limiting their economic opportunities and prospects for upward social mobility [65,66,67]. Within the housing market, stronger discriminatory measures were used against Aas [65,67]. In addition, many white people responded aggressively to the growing AA community by defending their homes with violence, intimidation, or legal tactics [65,67]. Other white people migrated to the suburbs in what became known as the “white flight” [65,67]. Despite these struggles, the Great Migration to Northern and Western cities provided the opportunities for AAs to form a new base of political power without the imposed restrictions of Jim Crow to fight against racial segregation through the growing Civil Rights Movement. 

The Civil Rights Movement gained traction by the 1950s with the lynching of the 14-year-old Emmett Till. The decision of Emmet Till’s mother to have an open-casket funeral sparked an emotional reaction that inspired the AA community to address racial segregation [68]. Soon after, Rosa Parks, Martin Luther King Jr., and other civil rights activists gained prominence in the activism against racial segregation and injustice. The resulting Civil Rights Movement ultimately led to the march on Washington for jobs and freedom, which put pressure on Presidents John F. Kennedy and Lyndon B. Johnson. In the end, Johnson backed the Civil Rights Act of 1964, which outlawed discrimination in places of public accommodation, the workplace, and labor unions, as well as the Voting Rights Act of 1965, which widened federal control over states to ensure Black political participation by safeguarding voter registration and elections.

### African Americans—A Historical Background on Systematic Inequalities

Despite the significant strides made by the AA community toward racial equality, several lines of systemic racism and socioeconomic inequalities continue to impact the health of the AA community [69]. Health outcomes for AA patients are poorer in several categories than for non-Black individuals. For example, AA women have a higher rate of maternal mortality due to pregnancy complications than White women, a disparity that persists even when patient factors such as age and income level are taken into account [70]. In 2018, Black infant mortality was 10.62 per 1000 live births, compared to 4.68 per 1000 live births for White newborns. The evidence suggests that when a physician is Black, AA patients seek out more preventative care [70]. In addition, black newborns cared for by Black physicians had a 40% lower death rate than when they were cared for by White physicians [70]. The fact that just 5% of physicians in the US are African-American further reinforces ongoing health inequalities. As such, it remains important to understand the historical basis by which the medical community has fostered health inequalities in the context of America’s deep legacy of white supremacy, slavery, and systemic racism.

For nearly 400 years, there has been a presumption of AA intellectual and biological inferiority amongst the broader American public and academic community [61,71]. The British colonization of North America, Western medicine, and biology (then a branch of the natural sciences) had all contributed to the development of the racial inferiority of AA [61,71]. By the 20th century, racial theories on AA and other minority groups were essential components of medical education, which often justified a lack of health provisions for AAs [72]. Despite being sworn to uphold the highest standards of professional ethics and humanitarianism, both European and American doctors contributed to the horrific conditions and exploitations of Africans both presently and through the transatlantic slave trade [72]. It was during this period that physicians introduced the idea that AAs had poorer health compared to white people. All these elements contributed to the pattern of subpar, inconsistent, or unavailability of health care for AAs during slavery, both after reconstruction and in the present [69,72]. The promise of liberation from slavery did not afford AAs equal status, especially concerning healthcare access. Although there have been health disparities in the AA community, excess deaths among the AA were only systematically studied with the publications of both the Heckler Report in 1985 as well as unequal treatment by the Institute of Medicine in 2002 [73]. Since then, disparities have been in almost all areas of medical care concerning the AA community and other minority groups. 

Therefore, the historical legacy of racial inferiority and systemic racism has had a disastrous impact on how AAs receive healthcare and interact with healthcare staff, particularly with the COVID-19 pandemic [72,74]. A recent survey study by the Pew Research Center showed that the beliefs of AAs concerning their overall health remain sharply divided. Specifically, 47% of AA respondents stated that their health has improved over the past 20 years, while 20% believe that it has worsened [74]. The main factor that AAs believes are responsible for their poorer health outcomes includes less access to high-quality medical treatment [74]. In addition, a large proportion of AAs also believe that other factors, such as poor environmental conditions in AA communities, hospitals, and medical facilities, contribute to the divide in healthcare outcomes [74]. A majority of AAs (51%) believe that previous medical issues are a key factor in their lower health outcomes. Furthermore, 47% of AA respondents believed that AAs were more likely to work in jobs that increased their risk for health problems. In addition, 52% claimed to be living in neighborhoods with more environmental health [74]. The majority of AAs had favorable opinions of the most recent care they received; however, the majority (56%) claimed to have encountered at least one unfavorable incident, such as needing to raise their voices to receive the right care and being treated less respectfully than other patients [74]. 

The systemic injustices faced by AAs within the healthcare community exemplify deep and unaddressed racial injustices since slavery. As manifestations of structural, cultural, and interpersonal racism adjust to changes in technology, cultural norms, and political events, the study of modern racism and its effects on health is complicated. This corpus of study exemplifies the various ways that the greater social environment may influence one’s health and health disparities. As such, there is an urgent need, especially in the AA community, to increase the focus on discovering the best strategies to lessen and eradicate the detrimental impacts of racism on health. Therefore, it is essential to recognize and effectively address how racism impacts health, particularly concerning AD and dementia care in the AA community.

## 4. Risk Factors/Susceptibility of AD in African Americans

Estimates of the prevalence of Alzheimer’s disease in African Americans differ significantly from those of white people, ranging from 14% to approximately 100% higher [75]. However, this increased prevalence of AD in AA is not well understood. Several genes have been identified that are uniquely found in the AA community compared to white people (Table 3). A recent meta-analysis by Reitz et al. showed that two lipid metabolism-related genes, apolipoprotein E (APOE) and ABCA7, are the primary heritable causes of AD in AA [76,77,78]. Specifically, the APOE allele was linked to a 2–3 times higher risk of AD in heterozygotes and a 12 times higher risk in homozygotes among white people [79]. However, the APOE allele is more common in AA, and homozygosity is strongly linked to AD in AA [77,79,80,81]. Alzheimer’s disease patients who had a homozygous mutation in both APOE alleles scored worse on difficult memory discrimination [82].

One of the most potent genetic risk factors for AD is the APOE allele [83]. Specifically, APOE has been associated with episodic memory-related impairment [79,84,85,86]. APOE has three different isoforms that are APOE2, APOE3, and APOE4, which differ only by two residues. The most significant genetic risk factor for AD is APOE 4, while APOE3 and APOE 2 have opposing effects that are neutral and protective, respectively [87]. One of the strongest risk factors for cognitive decline, particularly in those of European ancestry, is the APOE4 allele [88]. This allele is more prevalent in people of African ancestry, but in this population, has a less significant—yet still significant—relationship with cognitive deterioration [89]. By controlling lipoprotein uptake and modulating amyloid clearance, APOE controls lipid metabolism in the brain [90]. Studies have shown a prevalence of the APOE2 allele in AAs and a favorable lipid profile in APOE2 carriers, which may account for the protective impact of APOE2 observed in African Americans, whereas in Caucasians, the lack of such a protective effect may be due to APOE2 allele’s inability to affect the lipid profile [91]. The development of AD has been linked to both abnormal cholesterol processing and Aβ aggregation [80,90]. One of the first brain areas to be affected by AD is the medial temporal lobe [79,84,85,86]. In both rodent and human models, the APOE genotype and amyloid-induced synaptic pathology have been linked to accelerated rates of AD pathology in the medial temporal lobe [83,92].

Subsequent studies showed that there were APOE-related deficits in memory discrimination in a group of elderly AAs with normal cognitive function, which was accompanied by hyperactivity in the left dentate gyrus, CA3, and the CA1 regions of the hippocampus [81,93,94,95]. This finding may indicate that APOE-related hippocampal dysfunction can manifest in healthy older AA [81,93,94,95]. Therefore, it may be an indicator of future disease status, even though the overall impact of APOE on AD outcomes in AA is still unknown [81,93,94,95]. However, the findings of heterozygotic carriers are ambiguous, with some research indicating that APOE may have a less significant influence on AD outcomes among groups of African heritage, such as AA [93,95]. However, it is yet unknown how genetic risks interact with environmental and behavioral risk factors, as well as how these variables affect AD biomarkers in AA. There is also a lack of knowledge on the neurological bases of cognition in older African Americans and how these bases link to genetic risk factors for AD. Despite the inconsistent nature of these results, African Americans have a higher chance of developing late-onset AD [78].

In addition to APOE, recent studies have shown that the ATP-binding cassette transporter protein, ABCA7, has been identified as a unique genetic risk factor for AD in AA [18,78]. The ABCA7 is an integral transmembrane adenosine triphosphate-binding cassette transporter that mediates the metabolism of high-density lipoproteins using cellular lipid and helical apolipoproteins [18,78]. In addition, the ABCA7 transporter participates in apolipoprotein-mediated phospholipid and cholesterol efflux from cells by binding to apolipoprotein A1 [18,78]. Similar to APOE, this gene controls the balance of phospholipids and cholesterol in the brain and peripheral tissues [18,78]. Mutations in the ABCA7 are associated with an increased risk of AD which is comparable to the increased risk observed in AA-carrying mutated APOE alleles [18,78,96]. It is believed that the increased risk imposed by ABCA7 mutations is associated with APP processing and the suppression of Aβ clearance [96]. The further histopathological analysis found that mutations in the ABCA7 could facilitate increases in Aβ in the cortical regions of the brain, particularly in the medial temporal lobe and entorhinal cortex [20,97,98]. Further analysis showed that mutations in ABCA7 disrupted connectivity and synchronization between the entorhinal cortex and the medial temporal lobe regions within the hippocampus [99,100]. Given the overlapping functions of ABCA7 and APOE, it was suggested that there might be similar increases in AD risk in other minority risk groups.

Recently, it was shown that aerobic exercise supports brain lipid homeostasis and reduces the buildup of Aβ deposits [101,102,103]. Recent research has also suggested that higher levels of aerobic fitness might lessen the negative effects of polygenic susceptibility associated with AD, which is generated from genes associated with lipid homeostasis, such as APOE and ABCA7 [80]. Additionally, it has been discovered that in African Americans, the APOE genotype affects the exercise-related upregulation of the brain-derived neurotrophic factor (BDNF), a gene linked to neuroplasticity and hippocampal volume [104]. Non-carriers experienced a significant increase in BDNF levels after 6 months of exercise, whereas carriers did not [105]. Therefore, there may be additional interventions that be utilized to modify the expression of APOE and ABCA7 to prevent or reduce cognitive deficits in AA patients at risk for AD. 

### 4.1. Lifestyle, Diet, and Environmental Factors on AD/ADRD

**Figure 2:**
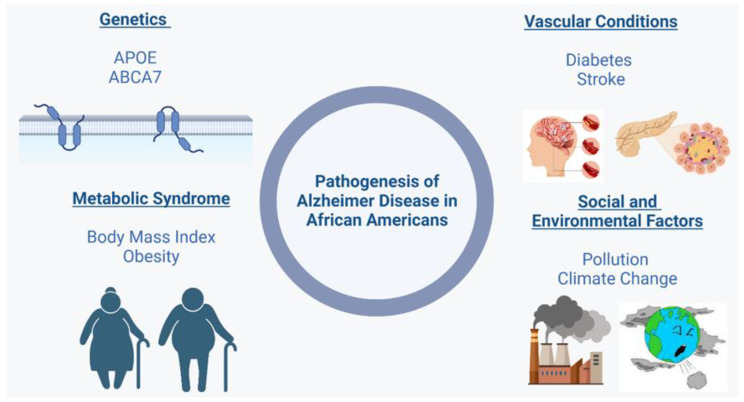
Pathogenic mechanisms of Alzheimer’s Disease in African Americans including genetic factors, vascular conditions, metabolic syndromes, and social and environmental factors.

In addition to genetic factors, other risk factors, such as lifestyle, diet, and environmental factors, have also been associated with the increased prevalence of AD/ADRD in the AA community, as shown in Figure 2. A study by the Commission on Dementia Prevention, Intervention, and Care showed that healthy nonsmoking, regular exercise, limited alcohol use, adequate sleep, and high-quality diets were protective against the onset and progression of AD [106]. The progression of cognitive decline and AD/ADRD correlates with classic cardiometabolic risk factors, such as a sedentary lifestyle, central obesity, dyslipidemia, insulin resistance, hypertension, diabetes, and cardiovascular disease. On the other hand, calorie restriction, dietary elements that are high in antioxidants, and certain dietary patterns may slow the progression of metabolic and neurodegenerative illnesses. The immune system is known to be regulated by nutritious foods high in antioxidants and anti-inflammatory characteristics. Furthermore, these foods may alter the neuroinflammatory processes implicated in the evolution of cognitive impairment and AD [107].

These nutritional supplements and eating habits might be effective strategies for preventing cognitive decline or delaying the development of AD/ADRD [108]. Numerous dietary elements have been researched for their effects on health and illness, including omega-3 fatty acids, nutraceuticals, minerals, micronutrients, and vitamins [109]. These dietary changes have been shown to have a positive impact on the pathophysiology of diseases, including diabetes, obesity, cardiovascular disease, and cancer, among others. Dietary changes affect several biological processes, including Aβ formation, tau hyperphosphorylation, oxidative stress, and epigenetic changes. The immune system is known to be regulated by nutritious foods that are high in antioxidants and which may alter the neuroinflammatory processes implicated in the evolution of cognitive impairments and AD [107]. These nutritional supplements and eating habits might be effective strategies for preventing cognitive decline or delaying the development of AD [108]. Numerous dietary elements have been researched for their effects on health and illness, including omega-3 fatty acids, nutraceuticals, minerals, micronutrients, and vitamins [109]. These dietary changes have been shown to have a positive impact on the pathophysiology of diseases, including diabetes, obesity, and cardiovascular disease.

Any movement made by skeletal muscles that require energy expenditure is referred to as physical activity [110]. Physically active people are generally healthy and free of various ailments [111]. This type of preventative action may significantly reduce the burden of illnesses that are linked to a certain lifestyle [112]. Physical exercise can reduce mitochondrial dysfunction by activating several transcription factors that are involved in bioenergetic processes, hence lowering the occurrence of a wide range of cardiometabolic and neurodegenerative disorders [113,114,115]. Regular exercise improves the health of the mitochondria in the skeletal muscles by activating a variety of cell signaling pathways [113,114,115]. It is well-recognized that exercise regulates blood sugar levels and body weight, keeping blood pressure stable, lowering dyslipidemia, and enhancing the health of the bones and muscles. Another study showed that transgenic mice brains accumulated fewer misfolded proteins and showed less cognitive deterioration [113,114,115].

Both animal and human studies have shown that physical activity enhances cognitive skills and causes the brain to become more malleable [116,117,118,119,120,121]. Through the actions of PGC-1 and SIRT1, a nicotinamide adenine dinucleotide (NAD)-dependent deacetylase, physical exercise regulates the cellular energy balance [116,117,118,119,120,121]. Exercise or calorie restriction depletes energy and raises the AMP/ATP ratio, which in turn causes the cell’s AMPK protein to become active. Subsequently, PGC-1 is then stimulated by these processes through phosphorylation, which eventually leads to the induction of mitochondrial biogenesis [116,117,118,119,120,121]. The age-related reduction in muscle mass and activity is a common phenomenon. Regular exercise prevents the degradation of muscles caused by aging and encourages healthy aging [116,117,118,119,120,121]. Another potentially beneficial non-pharmacologic strategy for slowing down the aging process of the brain is calorie restriction, which enhances metabolic health [116,117,118,119,120,121]. Calorie restriction works by counteracting the negative effects of reactive oxygen species and oxidative damage [116,117,118,119,120,121]. According to recent studies, long-term calorie restriction dramatically lowers levels of Aβ and beta-secretase in female Tg2576 mice and prevents the development of AD pathology [116,117,118,119,120,121]. Multiple metabolic variables that are related to the pathogenesis of cardiometabolic illnesses are said to be improved by calorie restriction in humans [116,117,118,119,120,121].

### 4.2. Lifestyle, Diet, and Environmental Factors on AD/ADRD in African Americans 

Given the importance of lifestyle factors and AD/ADRD, several studies have examined the lifestyle and diet factors that can contribute to the increased risk of AD among AAs. A previous study by Hossain et al. showed that a healthier diet among AA patients was associated with an improvement in cognitive performance [122]. In addition, differences in obesity and other diet-related chronic illnesses among AAs may be influenced by the targeted marketing of high-calorie foods and drinks to AA communities as opposed to more wholesome diets. Studies revealed that African Americans were routinely exposed to food distribution and marketing patterns that had a comparatively higher potential for negative health impacts than white people.

As such, the contribution of a person’s diet to their risk for AD/ADRD has gained further traction in the medical literature [123]. The Mediterranean diet, Dietary Approaches to Stop Hypertension, and the Mediterranean-DASH Intervention for Neurodegenerative Delay diets, which include berries, vegetables, and fish, have all been linked to slower cognitive decline as well as improving type 2 diabetes, obesity, and coronary artery disease [124,125,126]. It is believed that a diet that is higher in plant content slows cognitive decline by improving oxidative stress, inflammation, and vascular damage in the brain [124,125,126]. A study by Liu et al. examined whether there was an association between the rate of cognitive decline among AAs and white adults who consumed a plant-based diet [127]. The study used a total of 3337 patients, of whom 60% were AAs, to assess the rate of decline in global cognition, perceptual speed, and episodic memory [127]. The study found that a higher healthful planet diet index was associated with a slower rate of decline in global cognition, perceptual speed, and episodic memory among AA participants but not white participants [127]. Liu et al. believed that the greater benefits of a plant-based diet could be related to helping reduce the severity and onset of hypertension, diabetes, and stroke among AA [127]. Therefore, improvements in cardiovascular health may indirectly improve the onset and progression of AD among AAs who consume a plant-based diet [127]. In addition, it is believed that adherence to a healthy lifestyle, such as non-smoking, exercising, being cognitively active, having a high-quality diet, and limiting alcohol use, in addition to a plant-based diet, may be beneficial for AA patients who are carriers of the APOE allele [128].

### 4.3. Prevalence of AD in African Americans

**Figure 3:**
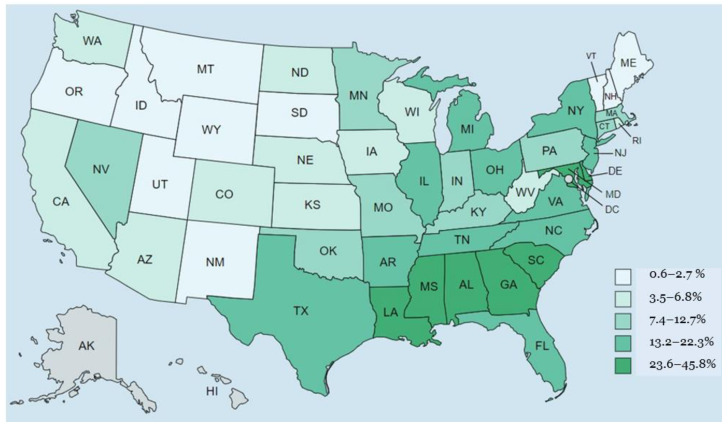
The figure shows the population distribution of African Americans in different states of the US. The population data are obtained from US Census 2021.

According to the US Census Bureau, the US population, excluding people who identify as bi- or multiracial, is estimated to be composed of 76.5% white and 13.4% AAs. The AA population distribution in different states of the US is presented in Figure 3. In comparison to other racial groupings, AAs had the greatest reported risk of ADRD among persons 65 years and older [129,130,131]. For instance, empirical estimates indicate that the prevalence of AD in Black people is highly variable, ranging from 14% to 500% higher than that of white people [129,130,131]. It is believed that several factors contribute to this discrepancy in the risk of AD among AAs, including genetics, lifestyle factors, socioeconomics, and other comorbidities (e.g., hypertension, diabetes mellitus, stroke, myocardial infarction, and congestive heart failure) [132,133,134]. Other sociological factors, including healthcare access/insurance coverage, systemic racism, clinical presentation, and the timing of diagnosis, further exacerbate these disparities in dementia care [135,136,137,138]. Furthermore, the clinical presentations among AA patients with dementia typically include neuropsychiatric symptoms, such as hallucinations, delusions, agitation, aggression, apathy, depression, and insomnia, that are reported less frequently in other ethnic and minority groups [139,140,141,142,143]. Compared to white people, AAs may display different clinical signs of Alzheimer’s disease due to their tendency to present at a younger age and with more severe symptoms [144]. This is consistent with the studies that AAs are less likely to seek medical care and when they do, present more severe symptoms [145]. In addition, AAs are also less likely than white people to obtain Alzheimer’s medications such as acetylcholinesterase inhibitors or memantine [146]. Therefore, further research on how such discrepancies affect the prevalence of cognitive diagnoses related to AD is required, given the well-documented health disparities that exist between racial groups in the US. 

Beyond the apparent racial disparities in the AA community, it is important to conduct more research to better understand the potential confounders of dementia diagnosis. African Americans’ participation in research studies is still less frequent than white people. In addition, lower participation rates have been reported among AAs for clinical trials [72]. The reasons for this lack of participation in clinical studies include study designs, logistical issues, inadequate health literacy, sociocultural variables, and other attitudes that discourage research engagement [72]. However, the biggest barrier to AAs participating in research is mistrust of academic and research organizations and investigators, which is based on historical incidents (e.g., Tuskegee) [72]. This mistrust among AAs towards healthcare professionals reflects ideas and attitudes of technical and interpersonal inadequacy, a focus on profit by doctors, and expectations on the experiments they participate in. AAs are also more likely than white people of the same age, education, and gender to think that any research they participate in would be used to support unfavorable racial stereotypes or not benefit the AA community [72]. Finally, researchers frequently restrict minority involvement because they are less likely to encourage minority patients to participate in clinical trials. However, other members of the AA community appreciate clinical research and are open to the idea that it may lead to the development of new and improved therapies for both them and the wider AA community. It is believed that racial differences in cognitive test performances may explain this discrepancy as older AAs tend to perform more poorly on cognitive tests compared to older white people [147]. As a result, these discrepancies pose a challenge for identifying dementia in older AA patients since cognitive performance tests are still the main criterion for diagnosing AD. It is hypothesized that using cognitive performance tests at a single point in time may lead to an overdiagnosis of AD in AA. This is despite AAs with Alzheimer’s disease showing a slower decline and longer survival rate compared with white people [144,148]. When it is possible, it is preferable to look at how cognitive function has changed over time.

The difficulty of assessing performance based on a single moment in time is alleviated by utilizing longitudinal data, where cognition is tested over numerous time points, given that the progression of Alzheimer’s disease involves a steady deterioration in cognitive function. Furthermore, the cognitive performance between white people and AAs shows little variation [149]. Multiple studies have shown that performance on neuropsychological tests is frequently influenced by educational and cultural experiences [150,151,152,153]. Clinicians and researchers may occasionally rely on the use of race-based norms, which are normative criteria for neuropsychological tests modified by race. These norms are typically developed by testing a sizable representative sample of individuals belonging to the same racial group (either nationally representative or representative of the local setting), who vary along a range of characteristics, therefore, it is important for neuropsychological tests to assess and interpret test performances for a particular racial group.

However, even though race-based norms are useful for improving diagnostic precision in clinical settings, their application makes several biological assumptions about the construction of race that could result in incorrect and potentially harmful interpretations of underlying racial differences [153]. The application of these principles also makes it hard to comprehend the underlying causes of such inequalities. Adjusting for elements that could have an impact on performance in cross-sectional assessments is a second method for addressing test-performance biases. As a result, there remain significant gaps in the variables that affect AA’s risk of developing AD. Several other factors can affect test performances among African Americans and are not typically taken into account when interpreting levels of cognitive impairment, especially in studies that compare African American-individuals to white individuals [154,155,156,157,158]. This is true even when adjustments are made for the factors that make the measures less valid in African American individuals. However, the field has had difficulty identifying reliable indicators of educational quality, and these indicators are rarely applied to make it easier to interpret test results in a clinical setting [144].

### 4.4. Status of Caregiving in AA and AD/ADRD 

Outside of the Alzheimer’s Association survey study, other research studies have found similar challenges among AA caregivers of AD/ADRD patients (Table 4). Anyone who offers care to someone who requires additional assistance, such as individuals with AD and ADRD, is referred to as the caregiver [159]. Within any caregiver–patient relationship, there are five main elements of interactions that this relationship provides to AD/ADRD patients, which include: family traditions, emotional connections, family values, a helping network, and expectations of family roles [160]. Compared to other caregiving groups, AA caregivers prefer to concentrate on the personality traits of the care receivers that remain instead of lamenting the losses brought on by dementia [161]. Furthermore, families in African American communities are convoluted networks of functional relationships, shared beliefs, affiliations, geography, and blood links [160]. Multiple connections, such as those between couples, children and parents, extended relatives, and friends, commonly make up families. Particularly among African American households, there may not be a single primary caregiver; instead, a team of persons manages the care of the AD/ADRD patient [160]. The majority of AA caregivers mention difficulties such as financial limitations, safety worries such as roaming, and difficulties with giving physical care [162]. Similarly, AA caregivers described stressful, unfavorable elements of providing care, although they achieved relatively high scores on quantitative measures of the quality of life [160]. Furthermore, AA caregivers describe difficulties with their personalities and with the dynamics of their evolving relationships with the AD/ADRD patient. Compared to white caregivers, fewer AA caregivers reported being affected by verbal hostility from AD/ADRD patients. It is thought that AA cultural values of familism and filial piety may have an impact on how people perceive stress and cope [160]. Compared to AA caregivers, white caregivers experienced greater sadness at higher levels and a decline in life satisfaction with time [163]. Physical problems reportedly became worse over time, according to both groups of caregivers. These findings point to deteriorating problems for many white caregivers over time.

However, they are still susceptible to long-term increases in physical symptoms [163]. Furthermore, AA caregivers exhibit more resilience on measures of depression and life satisfaction. When compared to white caregivers, AA caregivers were also less likely to decide to discontinue treatment at the time of death, less likely to have their loved one pass away in a nursing facility, and they also expressed less acceptance of the relative’s passing and a stronger sense of sorrow [164]. Despite these differences, both white people and AAs reported experiencing similar barriers to the cost of dementia care, a lack of family agreement about what to do, and transportation [165].

One common coping mechanism that showed positive effects in reducing AA caregiver burnout was having a strong faith background or community to discuss their struggles with and having the extra social capital to help with their loved ones’ care [166,167,168,169]. According to research on educational sessions held by faith-based organizations, these events helped AA caregivers (a) educate others on the pathophysiology of ADRD, (b) identify and then discuss risk-reduction strategies, and (c) emphasizes resources for families impacted by AD [168,169]. Additionally, culturally relevant instruction can help AAs become more literate in AD treatment and management. This research may point up methods to improve the teaching material and act as a manual for creating faith-based community initiatives that are appropriate to local cultures [168,169].

**Table 4:** AA caregiving status studies summary.

Author, Year	Study Type	AA Sample Size Caregivers	Results
Lindauer et al. [161], 2015	Interview	22	The findings of this study imply that rather than lamenting the losses brought on by dementia, AA caregivers frequently concentrate on the personality traits of the care receivers that remain.
McLennon et al. [170], 2020	Interview	12	AA caregivers described worries regarding immediate and long-term behavioral difficulties, family tensions, and isolation was voiced. They talked about incorporating care-related activities into daily life by learning to cope with caregiving tasks with acceptance, gratitude, and humor. Religious convictions, receiving support from others, and convictions about family obligations were additional ameliorating elements that were described in the context of family culture and values.
Roth et al. [163], 2001	Interview	197	Compared to AA caregivers, white caregivers throughout time experienced higher levels of heightened sadness and falling life satisfaction. The number of physical complaints in both groups of caregivers were reported to increase over time. AA caregivers have more resiliency on depression and life satisfaction scales, but they are still susceptible to long-term increases in somatic symptoms.
Owen et al. [164], 2001	Interview	63	Compared with white caregivers, AA caregivers were less likely to make a decision to withhold treatment at the time of death, less likely to have their relative die in a nursing home, and reported less acceptance of the relative’s death and greater perceived loss
Disbrow et al. [165], 2021	Interview	46	Barriers to care were similar across racial groups and included cost, a lack of family agreement about what to do, and transportation. Health literacy was a main barrier to caregivers regardless of race Many AA caregiver participants received information about ADRD from the television. However, AA caregivers found it difficult to separate evidence-based information from misleading advertisements
Spurlock et al. [166], 2005	Interview	71	AA caregivers reported a higher level of spiritual well-being than Caucasian caregivers and a lower level of caregiver burden
Heo et al. [171], 2013	Interview	211	AA caregivers demonstrated that higher religious coping resulting in lowering caregiver burden appraisal and thereby reducing depression
Epps et al. [172], 2020	Training Intervention	202	Most participants were adults who identified as AA (91%), female (75%), and were uninformed (66%). Many attendees identified themselves as clergy, members of the local community or church, caregivers, or medical professionals. Participants said the information was relevant to themselves or their families.
Wilks et al. [169], 2018	Interview	230	Spiritual support positively and significantly impacted resilience among both groups, slightly stronger among AA caregivers
Knight et al. [173], 2000	Interview	41	African American caregivers were also younger and in poorer health: factors which tended to increase both burden and emotional distress outcomes.
Dilworth-Anderson et al. [174] 2005	Interview	48	African Americans had stronger cultural reasons for providing care than white people, education levels were inversely related to the cultural justifications for caregiving scale (CJCS) scores, and the influences of gender and age on cultural reasons were moderated by race.
Samson et al. [175], 2016	Interview	32	Knowledge deficits were acknowledged. AA caregivers felt blindsided when faced with an AD diagnosis and did not know what to expect. AA caregivers regretted the lack of long-term health, legal, and financial planning within their families, driven somewhat from a cultural emphasis on privacy, particularly surrounding medical issues. AA caregivers likewise found it difficult to identify help within their own communities.
Dilworth-Anderson et al. [176], 2007	Interview/Qualitative Study	303	Between 7 and 22% of all groups of AA caregivers reported receiving help from their places of worship in the form of emotional support. Between 78 and 86%, of all caregivers indicated that their spiritual beliefs helped them a great deal with giving care. The three most common benefits of spiritual care among AA caregivers were: strength to endure (45%), a sense of duty and reciprocity toward those who had cared for them (24%), faith for encouragement and inspiration (24%)
Turner et al. [177], 2004	Interview	88	Formal caregiving is complicated by the distrust that many AA caregivers hold toward the health care system, which has resulted from years of exclusion, racism, and discrimination.
Cothran et al. [178], 2022	Interview	21	AA caregivers experience significant stressors in caring for family members where the complex interaction of sociocultural and environmental features and the perceptions of resources influence their coping strategies.
Guest et al. [179], 2021	Intervention	87	Interviews with participants identified the training had impacts on their caregiving strategies, their quality-of-life, and the perceived quality-of-life of the individual in their care
Czaja et al. [180], 2013	Intervention	54	AA caregivers who received the intervention reported a decrease in burden, an increase in perceived social support, and positive perceptions of the caregiving experience. No effect was observed for depression. Most caregivers indicated that the intervention improved their caregiving skills and found the technology to be easy to use.
Brewster et al. [181], 2020	Interview	142	AA caregivers who received either Great Village or Great Village + exercise reported significant reductions in depressive symptoms and an improvement in mastery. AA caregivers who received only Great Village reported a reduction in anxiety. Receiving no intervention worsened caregiver burden.
Wells et al. [182], 2017	Randomized Control Trial	109	The most commonly reported caregiver problems fell into five major categories: (a) communication problems with care recipients, family members, and/or significant others, (b) problems with socialization, recreation, and personal enhancement time; (c) problems with physical health and health maintenance, (d) problems in managing care recipients’ activities of daily living; and (e) problems with care recipients’ difficult behaviors.

### 4.5. Caregiving and AD/ADRD 

The neurodegenerative disorders AD and ADRD cause cognitive impairment and make it difficult for patients to handle daily tasks. They require assistance in accomplishing their daily tasks. Professionals who have been compensated to help someone with their everyday requirements are considered formal caregivers. Contrarily, unpaid informal caregivers are frequently members of the family, close friends, and volunteers [183]. It has long been known that caring for an elderly person who has dementia can be a source of stress for family members. The effects of caregiving on self-reported emotional distress are extensively documented in the literature, primarily in white caregiving populations. Studies comparing the psychological well-being of African American and white dementia caregivers have shown mixed results. The results have ranged from demonstrating no discernible differences across groups to demonstrating improved psychological well-being among African Americans [184]. Therefore, African Americans who provide care for others gain from their ethnicity as a culture rather than from the combined impacts of being a member of a disadvantaged minority group and the stress of providing care [173].

A qualitative study was conducted to access how African American family caregivers of individuals with AD/ADRD cared for them in their homes. A purposive sample of 16 African American informal caregivers participated in an in-depth interview. A sense of commitment, a difficult road, sentinel incidents, and faith in God were the four themes that stood out. The results of the study showed that caregivers needed more help with the process of providing care and needed to be well-informed about the needs of caregiving. Informal caregivers lacked supervision, support, and knowledge. Nursing-related implications included a focus on family assessment, education, resource knowledge, and cooperation with medical teams [185].

### 4.6. Social, Cultural, and Religious Factors

The long-standing cultural practice of African American households when caring for dependent seniors was supported by various studies. The cultural motivations for providing care had to be understood considering racial and gender indoctrination. The social roles that people in a specific cultural group play, such as son or daughter or husband or wife, could also influence how those people interpret cultural expectations and obligations [174]. Also, the amount of support offered to caregivers is likely to be influenced by structural factors and cultural norms. African American caregivers could have unique needs for organizing and carrying out caregiver duties [175]. In contrast to caregivers from other groups, AA caregivers exhibit different patterns of health-seeking behaviors and responses to interventions, which are aimed at promoting caregiver well-being. These differences may go beyond potential differences in the perception of and responses to the challenges of caregiving. Compared to their Caucasian counterparts, older African Americans used less formal health services and were more dependent on home care [186,187].

Even though no known cure for ADRD exists currently, several non-medical lifestyle behaviors were advised for possible prevention, such as healthy illness management and general age-related well-being [188]. For African Americans, religion provides significant emotional support, and religious attendance or religiosity can often be associated with better health results, which is why sensitivity to spirituality as a component of their cultural context is required for providing care for African Americans. [189]. A study conducted by Dilworth-Anderson and colleagues [176] investigated if and how caregivers’ spiritual beliefs, involvement in their religion, and allegiance with it affected their ability to care for elderly relatives. According to their findings, between 7% and 22% of all caregiver categories (primary, secondary, tertiary, and tertiary only) said that their places of worship had assisted them. When church assistance was given, it was primarily psychological assistance (i.e., advice and encouragement). Between 78% and 86% of all caregivers reported that their spiritual convictions aided a lot of them in providing care.

### 4.7. Family Structure

Black Caribbeans and African Americans share many comparable life experiences in the United States, including attending the same schools, residing in segregated neighborhoods, and encountering prejudice [190,191]. To manage stressors and meet everyday obligations, African American families frequently participate in patterns of providing and receiving emotional and practical support (such as housework, childcare, transportation, and financial support) [192].

The literature review shows how cultural history and Afrocentric beliefs influence the parenting methods, behaviors, traits, and strengths of African American families. The idea that personal identification and functioning take place within families, communities, and fictive familial networks are examples of communalism and harmony, which are Afrocentric principles [193]. Because it differs from the standard American family structure, family life in the African American community has frequently been perceived incorrectly. The nuclear family, which consists of a husband and wife raising their children, is the standard way that family is defined. Different family compositions and family configurations are regarded as deviating from the nuclear family model. African American families frequently span multiple generations and are not just made up of blood relations or even members of the nuclear family [194]. In African Americans, immediate family members, acquaintances, neighbors, and churchgoers frequently make up extended family networks [195]. Depending on the presence or absence of parents, children, or other adult or child family members, the structure and makeup of an African American family might change [196]. The African American family, then, represents significant relationships and influences that extend beyond the mother, father, and kids to include grandparents, parents, siblings, and cousins of partners and children [197].

The family has been a crucial institution in the African American community’s survival: helping and supporting the weak has been a major motif [198]. The priority of blood links above all other relationships, including marriage, the development of extended family bonds, a significant emphasis put on children, and respect for the old are a few examples of cultural values associated with family survival. The way that African Americans define and experience caregiving has been impacted by these cultural ideals [199].

### 4.8. Interventions (Education; Telemedicine; Sociological; Psychological)

African American AD/ADRD family caregivers are underrepresented in intervention research, and there is conflicting data on both their mental and physical health results. These caregivers are more likely to suffer from morbidity and death due to the stress of providing AD/ADRD care as well as the well-established health disparities of African Americans [200]. More than any other demographic, African Americans have greater rates of dementia, such as Alzheimer’s and others that are related. By 2050, it was anticipated that the number of instances of Alzheimer’s disease and other dementias would have doubled, disproportionately affecting the African American community, recognizing the demand for culturally appropriate programming for Alzheimer’s patients, their caregivers, and individuals with associated dementias.

African American caregivers have distinctive symptom patterns and reactions to treatments that are intended to improve caregiver well-being. To investigate how racial and cultural concerns might be incorporated into a culturally sensitive intervention for AA dementia family caregivers, a study investigated qualitative focus group data from 32 AA caregivers. Caregiver suggestions for changes to an existing psychoeducation program were solicited via scripted questions regarding their experiences providing care. The tradition of family care, caring, and caregiver concerns, culturally appropriate care, and navigating without a map were the four main topics that emerged from the analysis. The promotion of self-care, the development of caregiver skills and knowledge, and consideration of the AA family and community as resources for care were suggested as elements of an educational program [175].

Few interventions specifically target family caregivers and do not address caregiver burden or readiness for home care, according to national guidelines, which consider family caregiver engagement to be potentially the most beneficial but least used intervention [201,202]. Although there is evidence of racial and ethnic differences in family caregivers’ experiences, it is unknown to what extent culturally appropriate caregiver interventions exist. The familism, language, literacy, elder protection, and logistical constraints can all be handled through cultural tailoring. The studies showed the need for additional caregiver interventions that thoroughly assessed the advantages of culturally appropriate care [203].

Due to problems such as cost, logistics, a lack of understanding about available resources, or insufficient support from others, available services and intervention programs for dementia caregivers have been frequently underutilized. Information technologies have the potential to lower these obstacles and make it easier for caregivers to obtain the assistance they require [180]. Studies have shown that older patients and African Americans are more likely to engage in telephone coaching contacts and have access to telephones compared to computers or Internet connections, which is a culturally sensitive concern for this intervention [204]. Telephonic interventions are chosen due to their low cost, convenience of use, and delivery of interventions.

A recent study reported the effect of a psychoeducational intervention on caregivers. The psychoeducational intervention aimed to enhance caregiver abilities and enhance the quality of life for both the patients and their caregivers. The purpose of the study was to evaluate the impact of the Great Village and the cultural adaptation of a psychoeducational intervention on caregivers who identified as African American in terms of depressive symptoms, anxiety, burden, and mastery. Great Village exercise recipients reported significantly fewer depressive symptoms and increased expertise. Caregivers who just used Great Village reported feeling less anxious. The caregiver burden increased when no intervention was provided. The study suggested that culturally appropriate treatments should be provided to African American caregivers to support their health and well-being and increase their competency in caregiving [181].

### 4.9. Alzheimer’s Association Survey on Racial Disparities in AD among U.S. Adults

Dementia care is not exempt from racial and ethnic inequities in health and medical care, as was seen during the COVID-19 pandemic [10]. Race, ethnicity, location, and socioeconomic levels all have a significant impact on stigma, cultural differences, knowledge, and understanding, as well as the capacity for patients to receive a diagnosis, management, and receive care and support services for dementia. Beyond clinical treatment, these discrepancies also affect the way that Black, Hispanic, Asian, and Native Americans are reflected in clinical trials for Alzheimer’s disease [10]. Systemic racism, health inequalities, and socioeconomic inequities all raise the risk of Alzheimer’s and dementia in underrepresented racial and ethnic groups. In addition, older Black and Hispanic Americans had disproportionately higher rates of Alzheimer’s disease and other dementias than older white Americans as well as higher rates of missed diagnoses. Racial and ethnic differences in caregiving for those with Alzheimer’s or other dementias also exist. These variations include the accessibility of support resources, the amount of time spent providing care, cultural beliefs about the hardship of providing care, whether social networks offer support, and the caregiver’s psychological health. 

In response, the Alzheimer’s Association, in conjunction with Versta Research, conducted a survey of 2491 U.S. adults and the current or recent caregivers of adults aged 50 or older with cognitive issues to better understand racial and ethnic attitudes and experiences regarding Alzheimer’s and dementia care in the United States [10]. The data were collected at the University of Chicago via the AmeriSpeak^®^ panel. The survey included 945 white respondents. Oversamples of 541 Hispanic, 515 Black, and Asian 412 Americans were weighted to reflect each minority group’s true population proportions to allow for accurate statistical analysis and reporting. For Native Americans, the same survey was administered to 406 Native Americans recruited online with sampling stratified and data weighted on gender, age, income, and education to match the U.S. Census Bureau data. The availability of care and support services, confidence in medical professionals and the healthcare system, involvement in clinical trials and research, and caregiver experiences were among the questions posed to respondents.

Among the respondents interviewed, 19% of Asian Americans, 18% of Hispanic Americans, and 36% of AAs believed that prejudice prevented them from accessing Alzheimer’s treatment for themselves or their loved ones. In addition, more than 42% of Native Americans and 34% of Asian Americans, and 33% of Hispanic Americans also reported having encountered discrimination when seeking medical attention. Furthermore, 50% of AA, 34% of Asian Americans, and 42% of Native Americans reported facing discrimination in their clinical encounters [10]. Among non-white caregivers, half or more reported encountering prejudice when navigating medical settings for their loved ones [10]. The main worry among non-white caregivers was largely focused on whether staff members or physicians would not listen to them because of their race, color, or ethnicity. Specifically, 42% of AAs, 31% of Native Americans, 30% of Asian Americans, and 28% of Hispanic caregivers expressed similar fears regarding healthcare staff. In contrast, less than 17% of white caregivers shared this opinion [10]. In addition, 41% of caregivers who offered unpaid care to an AA family member felt that their race made it more difficult for them to access high-quality medical treatment [10].

In addition, non-white people reported wanting their healthcare professionals to be aware of their particular circumstances and histories, although many were skeptical that they would have access to such professionals [10]. The survey of respondents found that the vast majority of non-white Americans, including Native Americans (92%), AAs (89%), Hispanics (85%), and Asian Americans (84%), believed that Alzheimer’s and dementia care providers must comprehend their ethnic or racial background and experiences when dealing with medical staff and addressing the needs of their loved ones. However, 63% of Asian Americans and 59% of Hispanics reported feeling confident about their ability to access culturally competent providers. Only 48% of AAs and 47% of Native Americans reported having similar perceptions of their healthcare providers [10]. Furthermore, AAs have little faith in scientific trials and have second-guessed the dissemination of new Alzheimer’s treatments. Compared to all other groups surveyed, AAs are the least likely to be interested in taking part in clinical trials for Alzheimer’s disease, with 62% of AAs believing that medical research is prejudiced against people of color. Only 53% of AAs believed that everyone would have access to a future Alzheimer’s cure, regardless of race, color, or ethnicity [10]. When asked if they would visit a doctor if they were having thinking or memory issues, Hispanic, Black, and Native Americans were twice as likely as white people to indicate they would not. In addition, 20% of Hispanics and AAs said they would be offended if a doctor recommended a cognitive test for themselves. Compared to other ethnicities, Hispanics, and AAs were less concerned about burdening their families if they were diagnosed with Alzheimer’s disease. Across all demographics, over two-thirds of caregivers reported providing care to AD patients as being very difficult to manage [10]. Given the challenges and disparities reported by AD caregivers, the Alzheimer’s Association further investigated these disparities among caregivers.

### 4.10. Alzheimer’s Association Survey on Racial Disparities among AD Caregivers

A survey of 1392 U.S. citizens by the Alzheimer’s Association was performed on those who now or recently provided unpaid care for an adult family or friend who was 50 years of age or older and was having symptoms associated with dementia [10]. The sample consisted of caregivers who identified as 313 white, 309 Hispanic, 305 AA, 301 Asian, and 154 Native American. Ten caregivers were identified as belonging to another racial or ethnic group. More than 36% of AA, 18% of Hispanic, and 19% of Asian Americans believed prejudice was a barrier to accessing Alzheimer’s and dementia care, according to the Alzheimer’s Association poll of U.S. adults [10]. They specifically anticipate being treated differently according to their race, ethnicity, or color. Among the different factors examined, affordability was one of the other perceived hurdles to treatment mentioned by survey participants, followed by a lack of adequate local health care (particularly among Black Americans and Asian Americans), a lack of good health insurance coverage, and a lack of family and social support.

In addition, other studies have found that caregivers reported issues with healthcare providers, including communication issues with care recipients, family members, and/or significant others, issues with socialization, recreation, and personal enhancement time, as well as issues with physical health and health maintenance, managing the care recipients’ activities of daily living, and difficult behaviors exhibited by caregiver recipients [182]. Fewer respondents believed that language was a barrier to obtaining dementia care, although almost 23% of Asian Americans and 17% of Hispanic caregivers reported similar observations. More than two-thirds of AAs stated it was more difficult for an AA to receive high-quality treatment for dementia or Alzheimer’s disease when questioned specifically about the influence of race or ethnicity on care quality. Likewise, two in five Native Americans and Hispanic Americans, as well as one-third of Asian Americans, reported that their race or ethnicity made it more difficult to obtain care [10].

The greatest hurdle, according to caregivers, is prejudice, which is cited as a barrier by 25% of AAs, 19% of Native Americans, 17% of Asian Americans, and 8% of Hispanic caregivers [10]. More than half of caregivers who identify as Native American, AA, or Hispanic reported that they had encountered racial prejudice when navigating medical facilities for the care recipient. For 47% of Asian Americans, the same was true. In addition, 41% of caregivers who offered unpaid care to AA people felt that their race made it more difficult for them to access high-quality medical treatment [10]. Overall, the main issue reported by most minority groups was the lack of being heard by employees or providers because of their race, ethnicity, or color, which was reported by 42% of African American caregivers, 3% of Native Americans, 30% of Asian Americans, and 28% of Hispanics caregivers [10]. Only 17% of white caregivers reported having similar experiences with healthcare providers. Compared to 11% of white caregivers, more than one in four non-White caregivers reported experiences with healthcare professionals treating them as if they were less intelligent. Additionally, at least 20% of caregivers who are not white claim to have received less respect and/or decency from their employers [10]. Most people who were caring for a non-white person agree that healthcare professionals must be aware of the racial or ethnic background and experiences of the person they are caring for [10]. 

Additional information was revealed by the Alzheimer’s Association study of caregivers, which showed that for many family members and friends who look after a loved one with dementia, the benefits of caring for them may help balance stress [10]. More than half of unpaid caregivers asked said they helped someone with personal care activities, including eating, dressing, and bathing. The proportion of caregivers who provided this type of care was highest among AA and Hispanic caregivers, followed by Asian Americans and Native Americans. Compared to other groups, AA (78%) and Hispanic Americans (83%) were less concerned about burdening their family if they were diagnosed with Alzheimer’s disease [10]. Nearly all caregivers agreed that providing care is rewarding, even though nearly two-thirds of caregivers say it is stressful. The results of the Alzheimer’s Association surveys show that there is still much work to be conducted in addressing health and healthcare disparities in the care of people with Alzheimer’s and dementia.

## 5. Recommendations from the Alzheimer’s Association Survey on Racial Disparities in AD and Caregivers

The results of the Alzheimer’s Association surveys show that there is still much work to be conducted in addressing health and healthcare inequities in the care of people with Alzheimer’s and dementia. Accelerating current initiatives to address socioeconomic determinants of health, reduce health inequalities, increase diversity in the healthcare profession, and train healthcare professionals are necessary to fulfill the requirements of an aging population of older persons from various racial and ethnic groups [10]. Greater efforts must be made to end discrimination and other forms of bias amid broader calls for social justice to guarantee that all Americans have access to high-quality dementia care and support services as well as opportunities to participate in Alzheimer’s research. In addition, the Alzheimer’s Association recommends that the healthcare workforce be trained specifically for a racially and ethnically diverse population of older adults with dementia. The Alzheimer’s Association also suggests engaging, recruiting, and retaining diverse populations in Alzheimer’s research and clinical trials (Table 5) [10]. 

At the provider level, cultural competency training gives specialists the knowledge and tools that are necessary to interact with dementia patients and caregivers from all racial and ethnic backgrounds in a way that is respectful of language and culture [10]. Furthermore, cultural competency supports the development of a diverse and inclusive workforce at the organizational level [10]. The Alzheimer’s Association suggests that organizations include culturally diverse staff that reflects the population served, including bilingual staff or interpreters, and provide patients with translated materials to present medical information that is sensitive to cultural norms [10]. Numerous initiatives have been made to include cultural competency in dementia care. One such instance is the memory support program (MSP) at Stanford Health Care, which guarantees continuity for patients and caregivers [10]. Cultural competency is a priority for other groups that work with medical professionals who can diagnose and treat people with dementia. The national culturally and linguistically appropriate services (CLAS) standards of the U.S. Department of Health and Human Services guide how to communicate with individuals from various ethnic groups in a way that is respectful of and sensitive to their culture. Together, these initiatives can help address the growing burden of Alzheimer’s disease and other dementias on disproportionately affected racial and ethnic groups.

## 6. Reducing Racial Disparities

The advantages of investigating racial and ethnic variations in medical care delivery and results, carrying out ethnographic studies to uncover the underlying factors, training personnel about the effects of bias and structural racism, and making conscious efforts to win patients’ trust are numerous. They also provide guidance on what might be required to advance past these infant steps and establish the pursuit of health equity as a standard practice. They consist of three primary aims: (1) Prioritizing the measurement of health disparities within institutions and among providers; (2) Building partnerships to enable patients to play a meaningful role in developing solutions; (3) Making racial equity a strategic priority [205]. Public reporting performance on racial disparity criteria has been implemented by healthcare groups which mandate that healthcare practitioners track racial and ethnic disparities in treatments for a variety of diseases [206,207]. Yet, many local, state, and federal governments, as well as the private sector, have not yet gathered, disseminated, or used data on racial health inequalities in ways that could spur change. Health systems can identify inequities in their current data even without public disclosures [205]. In addition, several healthcare companies collaborate with community advisory boards, gather patient feedback, and monitor outcomes to spot possible issues. Instead of producing uniform guidelines, this can result in personalized interventions. In most circumstances, healthcare staff use approaches toward the general population when discrepancies are observed between different ethnic groups and then assume that this leads to improved outcomes. Yet, doctors have a duty to recognize how distinct groups differ in terms of attitudes, access to care, and pertinent cultural issues and to take these quality initiatives into account [205]. Given the importance of addressing racial disparities with clear metrics, the following initiatives have been suggested to address racial disparities in healthcare (Table 6).

## 7. Conclusions

Alzheimer’s disease and ADRD are chronic illnesses that are highly prevalent in AAs due to multiple factors, including genetic mutations, modifiable and non-modifiable risk factors, and lifestyle. The prevalence of AD and ADRD among AAs is further exacerbated by centuries of systematic racism and disparities that continue to affect the AA community. As such, the contributions of race, ethnicity, location, and socioeconomic status all have a significant impact on the care and support services available for dementia among AA patients. Furthermore, disparities in healthcare are entangled with social, economic, and environmental variables that perpetuate disadvantages among different groups, particularly African Americans. As such, it remains important to understand how various racial and ethnic groups perceive, access, and experience healthcare. Considering the mounting data showing that AAs may be more susceptible to AD than white people, this demographic transition will create significant hurdles in providing adequate care from family caregivers. Compared to other ethnic groups, AA caregivers face significant hurdles in providing formal and informal caregiving to their loved ones with AD or ADRD. These issues are further exacerbated by caregiving dynamics among family members and society at large. Despite advances in understanding the pathophysiology of AD, the importance and challenges of caregiving have been largely overlooked concerning AD treatment. Furthermore, there have been fewer clinical trials or studies to assess interventions in AD caregiving that would be beneficial for AA or other ethnic groups with family members suffering from AD. Additional training in supporting caregivers and providing adequate training for healthcare staff to understand the unique challenges faced by the AA community would greatly improve caregiver burnout and the long-term outcomes of dementia care. 

## Figures and Tables

**Table 1:** Classification of dementias based on neuropathological features.

Dementia	Features
Frontotemporal Dementia (FTD)	- disturbances in personality, behavior, and language - focal degeneration of the frontal temporal lobes [45]
Lewy-body Dementia (LBD)	- Varying cognition with noticeable shifts in attention and alertness - recurrent visual hallucinations - spontaneous motor features of parkinsonism frequent, such as falls, syncope, - a brief loss of consciousness, - systematized delusions [46]
Multiple Etiology Dementia (MED)	- Alzheimer’s disease and cerebrovascular disease coexist - compromised memory - white matter lesions in brain imaging - localized neurological symptoms [47]
Vascular Dementia (VD)	- cerebral lesions of vascular origin that lead to varied cognitive symptoms [48] - atherosclerosis - cerebral amyloid angiopathy - lipohyalinosis

**Table 2:** Definitions for racial or minority groups from the U.S. Census Bureau *.

Race or Minority Group	Definition
White	A person with origins from any of the original peoples of Europe, the Middle East, or North Africa
Black or African American	A person with origins from any of the Black racial groups of Africa.
American Indian or Alaska Native	A person with origins from any of the original peoples of North and South America (including Central America) and who maintains tribal affiliation or community attachment.
Asian	A person with origins from any of the original peoples of the Far East, Southeast Asia, or the Indian subcontinent including, for example, Cambodia, China, India, Japan, Korea, Malaysia, Pakistan, the Philippine Islands, Thailand, and Vietnam.
Native Hawaiian or Other Pacific Islander	A person with origins from any of the original peoples of Hawaii, Guam, Samoa, or other Pacific Islands.
Multi-racial	Identifying as one of the above five major groups according to the U.S. Census Bureau

* https://www.census.gov/topics/population/race/about.html (accessed on 6 March 2023).

**Table 3:** Known genetic loci associated with AD in whites and African Americans [78].

White	African American
CR1 (complement activation)	CR1 (complement activation)
BIN1 (endocytosis/apoptosis)	BIN1 (endocytosis/apoptosis)
EPHA1 (nervous system development)	EPHA1 (nervous system development)
CD33 (immune system)	CD33 (immune system)
ABCA7 (lipid metabolism)	ABCA7 (lipid metabolism)
APOE (lipid metabolism)	APOE (lipid metabolism)
CLU (chaperone protein)	HMHA1 (cytoskeletal remodeling and cell spreading)
MS4A6A/MS4E4 (Induces anti-inflammatory neuroprotective phenotype)	GRIN3B (Encodes a subunit of the *N*-methyl-D-aspartate (NMDA) receptor)
CD2AP (Stabilizes the interaction between T cells to antigen-presenting cells)	
PICALM (Regulates APP internalization and subsequent Aβ generation)	

**Table 5:** Alzheimer’s Association Guidelines to address health disparities for Alzheimer’s caregivers [10].

Preparing the workforce to care for a racially and ethnically diverse population of older adults
Increasing diversity in dementia care.
Engaging, recruiting, and retaining diverse populations in Alzheimer’s research and clinical trials.
Train culturally diverse staff that reflects the population served.
Use interpreters and bilingual staff to overcome language barriers
Training for providers on the cultures and languages represented in the population
Patient materials and practice signage that is translated and sensitive to cultural norms.
Recognizing the changing racial and ethnic demographics of Alzheimer’s disease
Recognize and overcome implicit bias is another method that organizations are using to tackle disparities.
Train health care providers to screen, diagnose, and treat Alzheimer’s and dementia in this expanding racially and ethnically diverse population of older adults so that disparities are not perpetuated

**Table 6:** Strategies for combatting systemic racism in healthcare [205].

1. Examining institutional policies with an equity lens
2. Establishing accountability frameworks such as equity scorecards
3. Auditing medical school curricula for erroneous references to race
4. Reviewing clinical algorithms that erroneously rely on race
5. Investing in scholarships for students of color interested in health professions
6. Training leadership and staff in diversity, equity, inclusion, and antiracism principles
7. Creating real-time reporting initiatives to track and respond to racist or other discriminatory behavior
8. Reviewing vendor relationships to support Black and other minority-owned businesses
9. Creating more equitable workplaces, including efforts to build wealth and opportunities for advancement
10. Listening to and learning from patients and health care professionals of color

## Data Availability

Not applicable.
